# Clinical impact of changes in mitral regurgitation severity after medical therapy optimization in heart failure

**DOI:** 10.1007/s00392-022-01991-7

**Published:** 2022-03-16

**Authors:** Matteo Pagnesi, Marianna Adamo, Iziah E. Sama, Stefan D. Anker, John G. Cleland, Kenneth Dickstein, Gerasimos S. Filippatos, Riccardo M. Inciardi, Chim C. Lang, Carlo M. Lombardi, Leong L. Ng, Piotr Ponikowski, Nilesh J. Samani, Faiez Zannad, Dirk J. van Veldhuisen, Adriaan A. Voors, Marco Metra

**Affiliations:** 1 Institute of Cardiology, ASST Spedali Civili, Department of Medical and Surgical Specialties, Radiological Sciences and Public Health, University of Brescia, Brescia, Italy; 2 Department of Cardiology, University Medical Center Groningen, Groningen, The Netherlands; 3 Division of Cardiology and Metabolism, Department of Cardiology (CVK) and Berlin-Brandenburg Center for Regenerative Therapies (BCRT), German Centre for Cardiovascular Research (DZHK) Partner Site Berlin, Charité Universitätsmedizin Berlin, Berlin, Germany; 4 National Heart and Lung Institute, Royal Brompton and Harefield Hospitals, Imperial College, London, UK; 5 University of Bergen, Bergen, Norway; 6 Department of Cardiology, Attikon University Hospital, National and Kapodistrian University of Athens, Athens, Greece; 7 School of Medicine Centre for Cardiovascular and Lung Biology, Division of Molecular and Clinical Medicine, Ninewells Hospital and Medical School, University of Dundee, Dundee, UK; 8 Department of Cardiovascular Sciences, Glenfield Hospital, University of Leicester, Leicester, UK; 9 Department of Heart Diseases, Wroclaw Medical University, Wrocław, Poland; 10 Universite de Lorraine, Inserm, Centre d’Investigations Cliniques 1433 and F-CRIN INI-CRCT, Nancy, France; 11 Robertson Centre for Biostatistics and Clinical Trials, University of Glasgow, Glasgow, UK; 12 Stavanger University Hospital, Stavanger, Norway; 13 NIHR Leicester Biomedical Research Centre, Glenfield Hospital, Leicester, UK

**Keywords:** Mitral regurgitation, Heart failure, GRMT, Valvular heart disease, Mortality, Hospitalization

## Abstract

**Background:**

Few data are available regarding changes in mitral regurgitation (MR) severity with guideline-recommended medical therapy (GRMT) in heart failure (HF). Our aim was to evaluate the evolution and impact of MR after GRMT in the Biology study to Tailored treatment in chronic heart failure (BIOSTAT-CHF).

**Methods:**

A retrospective post-hoc analysis was performed on HF patients from BIOSTAT-CHF with available data on MR status at baseline and at 9-month follow-up after GRMT optimization. The primary endpoint was a composite of all-cause death or HF hospitalization.

**Results:**

Among 1022 patients with data at both time-points, 462 (45.2%) had moderate-severe MR at baseline and 360 (35.2%) had it at 9-month follow-up. Regression of moderate-severe MR from baseline to 9 months occurred in 192/462 patients (41.6%) and worsening from baseline to moderate-severe MR at 9 months occurred in 90/560 patients (16.1%). The presence of moderate-severe MR at 9 months, independent from baseline severity, was associated with an increased risk of the primary endpoint (unadjusted hazard ratio [HR], 2.03; 95% confidence interval [CI], 1.57–2.63; *p* < 0.001), also after adjusting for the BIOSTAT-CHF risk-prediction model (adjusted HR, 1.85; 95% CI 1.43–2.39; *p* < 0.001). Younger age, LVEF ≥ 50% and treatment with higher ACEi/ARB doses were associated with a lower likelihood of persistence of moderate-severe MR at 9 months, whereas older age was the only predictor of worsening MR.

**Conclusions:**

Among patients with HF undergoing GRMT optimization, ACEi/ARB up-titration and HFpEF were associated with MR improvement, and the presence of moderate-severe MR after GRMT was associated with worse outcome.

**Graphical abstract:**

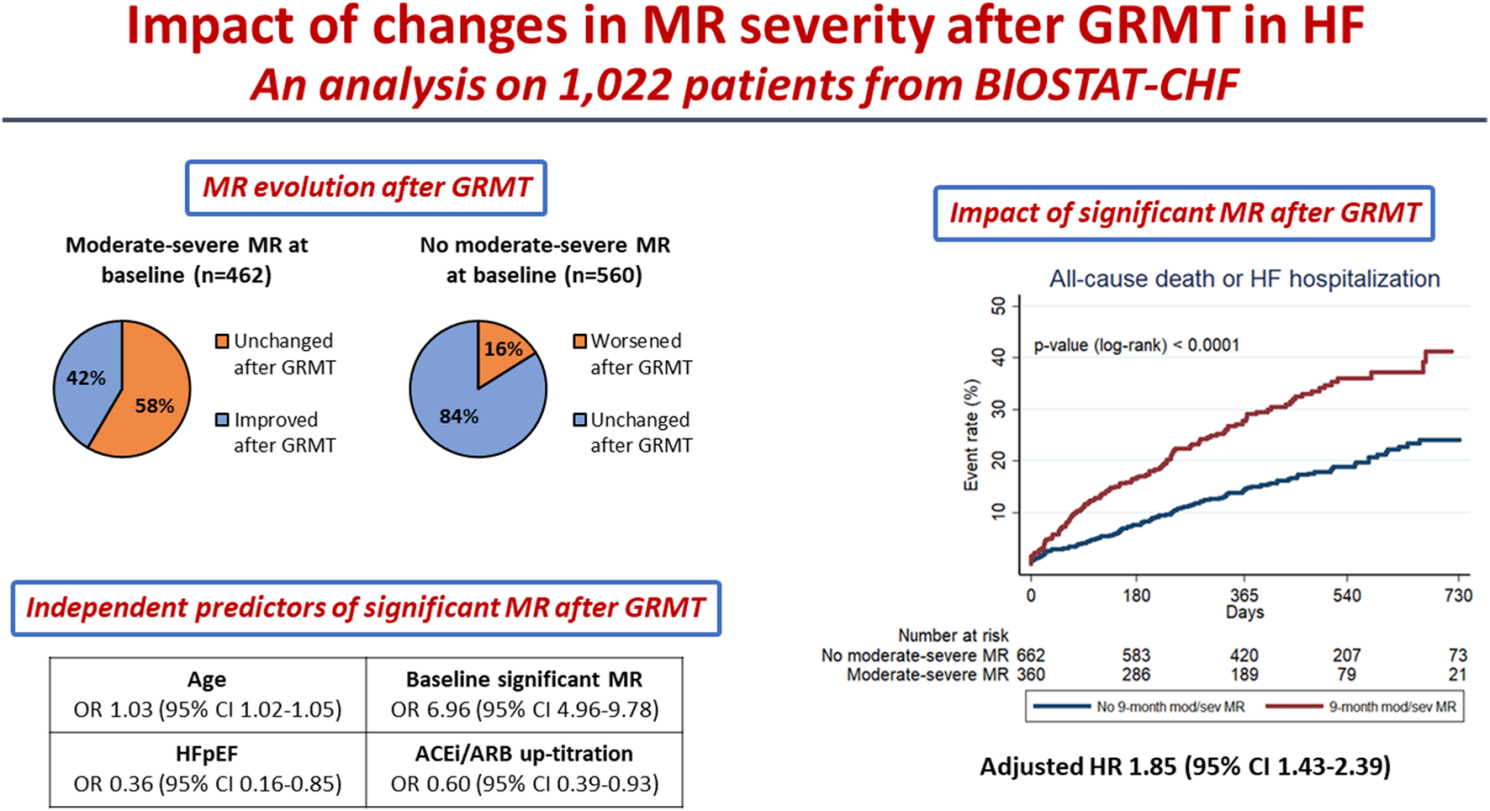

**Supplementary Information:**

The online version contains supplementary material available at 10.1007/s00392-022-01991-7.

## Introduction

Mitral regurgitation is the most common valvular heart disease in patients with heart failure (HF) [1–3]. It has a strong prognostic impact in both acute and chronic settings [4–17], and has emerged as a potential therapeutic target in HF patients [18, 19]. Medical therapy with β-blockers and angiotensin-converting enzyme inhibitors (ACEi), angiotensin receptor blockers (ARB) or angiotensin receptor neprilysin inhibitors (ARNI) is the mainstay of treatment in HF with reduced ejection fraction (HFrEF) [20–23]. Initiation and up-titration of these agents to target doses is recommended in current HF guidelines [20, 23], and represents a crucial step in HF management before evaluating interventional procedures. The Cardiovascular Outcomes Assessment of the MitraClip Percutaneous Therapy for Heart Failure Patients with Functional Mitral Regurgitation (COAPT) trial enrolled symptomatic patients with persistent MR despite attempted optimization of guideline-recommended medical therapy (GRMT) for HF, and demonstrated the superiority of percutaneous mitral valve repair plus GRMT compared to GRMT alone in a carefully selected population [19, 24, 25]. Of note, reduction in MR severity has been described in HF patients treated with β-blockers, renin–angiotensin–aldosterone system (RAAS) inhibitors or cardiac resynchronization therapy [26–31]. Hence, further assessment of changes in MR severity after GRMT optimization and an assessment of their impact on patients’ outcomes seem warranted.

The aim of this study was to evaluate the evolution and prognostic impact of MR after up-titration of GRMT in patients included in Biology Study to Tailored Treatment in Chronic Heart Failure (BIOSTAT-CHF), a prospective observational multicentre study enrolling patients with worsening chronic or new-onset HF undergoing GRMT optimization [32–34].

## Methods

### Study population

The index cohort of BIOSTAT-CHF recruited 2516 patients from 69 centres in 11 European countries between 2010 and 2014. Included patients had symptoms of new-onset or worsening chronic HF, with either a left ventricular ejection fraction (LVEF) ≤ 40% or B-type natriuretic peptide (BNP) > 400 pg/mL and/or N-terminal pro-BNP (NT-proBNP) > 2000 pg/mL and were treated with oral or intravenous furosemide ≥ 40 mg/day or equivalent at inclusion. Patients should not have been previously treated with ACEi/ARBs and/or β-blockers or should have received ≤ 50% of the target doses of these drugs at inclusion, with an anticipated initiation or up-titration of ACEi/ARBs and/or β-blockers by the treating physician. In the first 3 months (optimization phase), initiation or up-titration of ACEi/ARBs and/or β-blockers was specified according to the 2008 European Society of Cardiology (ESC) guidelines [35]. In the subsequent 6 months (stabilization phase), no further treatment optimization was required, except in case of changes in clinical status. At 9 month, a mandated follow-up visit was performed, including clinical evaluation and echocardiography. Subsequent follow-up was performed at 6 month intervals by means of clinical visit or phone contact, until the end of follow-up on April 1, 2015. The study was approved by the ethics committees of all participating centres and all patients provided written informed consent.

For the purposes of the present study, patients from the index cohort with available MR data at both baseline and 9 months (*n* = 1022) were included. The study flowchart is shown in Supplementary Fig. 1.

### Study definitions and endpoints

Patients underwent two-dimensional transthoracic echocardiography at baseline and at 9-month follow-up using a commercially available echocardiography (3.5 MHz probe). According to the study protocol, MR was evaluated using two-dimensional and color Doppler echocardiography [36], and the presence of moderate or severe MR (as compared to no or mild MR) was recorded. Left ventricular (LV) diameters, LVEF according to the modified Simpson rule, and left atrium diameter were also quantified and reported. Baseline clinical characteristics, quality-of-life (QoL) measures and laboratory data at inclusion and 9 months, and clinical outcomes at follow-up were also assessed.

The primary endpoint was the composite of all-cause mortality or HF hospitalization. Secondary endpoints were all-cause mortality, cardiovascular (CV) mortality and HF hospitalization as individual outcomes.

### Statistical analyses

Continuous variables are presented as mean ± standard deviation or median (interquartile range, IQR), as appropriate, and were compared with the unpaired Student’s *t* test or the Mann–Whitney *U* test, respectively. Categorical variables are presented as number and percentages and were compared with the *χ*^2^ test. Baseline clinical characteristics, echocardiography data, laboratory data, QoL indexes, primary and secondary endpoints were compared between patients with vs. without 9-month moderate-severe MR and between the following four groups defined according to baseline and 9 month moderate-severe MR: patients without moderate-severe MR at baseline and 9 months (unchanged); patients with moderate-severe MR at baseline and without moderate-severe MR at 9 months (improved); patients without moderate-severe MR at baseline and with moderate-severe MR at 9 months (worsened); and patients with moderate-severe MR at baseline and 9 months (unchanged). The Kaplan–Meier method (log-rank test) was used to evaluate the first occurrence of primary and secondary endpoint in patients with or without 9-month moderate-severe MR and in the four groups describing MR evolution (censoring follow-up at 2 years). Cox proportional hazards regression analysis was also performed to evaluate the prognostic impact of 9-month moderate-severe MR on primary and secondary endpoints. Univariable analysis and multiple multivariable models were performed to adjust the presence of 9 month MR for the several clinical, laboratory, and echocardiographic covariates of interest, including the previously validated BIOSTAT-CHF risk prediction models (model 5) [33]. Results of the Cox regression analyses are reported as unadjusted or adjusted hazard ratio (HR) and 95% confidence interval (CI). Multivariable binary logistic regression analysis was also performed to identify independent predictors of moderate-severe MR at 9-month follow-up. Variables with a univariate *p* value < 0.10 or variables judged to be of clinical relevance were included into the final multivariable model. Results of the binary logistic regression are presented as odds ratio with 95% CI. The *C* statistic and Hosmer–Lemeshow goodness-of-fit test were used to evaluate the discrimination, calibration and fit of the multivariable model.

All reported *p* values are two sided, and *p* < 0.05 was considered statistically significant.

Statistical analyses were performed using STATA version 13.0 (STATA Corp., College Station, Texas).

## Results

Among the 1,022 patients included in the present study (Supplementary Fig. 1), 462 (45.2%) had moderate-severe MR at baseline and 360 (35.2%) had it at 9-month follow-up after the optimization phase. Regarding the evolution of MR over time, MR severity remained unchanged between baseline and 9 months in 470 patients (46.0%) without, and in 270 patients (26.4%) with, moderate-severe MR. Conversely, MR improved from moderate-severe at baseline to no or mild MR at 9 months in 192 patients (18.8% of all patients or 41.6% of those with moderate-severe MR at baseline). Conversely, 90 patients developed new moderate-severe MR by 9 months (8.8% of all patients or 16.1% of those with no or mild MR at baseline).

### Patients’ characteristics

Detailed clinical, echocardiographic, laboratory, and QoL characteristics across the four groups defined according to baseline and 9-month MR are reported in Supplementary Tables 1 and 2. Baseline clinical characteristics among patients included as compared to those excluded from the study are reported in Supplementary Table 3.

Compared to patients with no or mild MR, patients with moderate-severe MR at 9 months were older, had lower body mass index, and were more likely to have a history of chronic kidney disease, prior cardiac device therapy, and worsening chronic HF as cause of the baseline visit (Table 1). These patients also had more advanced symptoms (NYHA class) at 9 months and lower systolic blood pressure at baseline and 9 months compared to patients with no or mild MR. The percentage of patients who achieved the ACEi/ARB target dose at 3 months and the mean ACEi/ARB optimal dose fraction at 3 months were lower in patients with moderate-severe MR at 9 months. These differences were not observed for β-blockers.

**Table 1:** Clinical characteristics in patients with vs. without 9-month moderate-severe MR

	Overall (*n* = 1022)	Moderate or severe MR (*n* = 360)	No or mild MR (*n* = 662)	*p* value
Age (years)	66.9 ± 12.2	69.2 ± 11.1	65.7 ± 12.6	** < 0.001**
Men	786 (76.9)	273 (75.8)	513 (77.5)	0.548
BMI (kg/m^2^)	27.8 ± 5.4	27.0 ± 4.6	28.3 ± 5.7	** < 0.001**
HF hospitalization in last year	284 (27.8)	112 (31.1)	172 (26.0)	0.080
Primary ischemic HF aetiology	442 (43.9)	168 (47.3)	274 (42.0)	0.105
Medical history				
Hypertension	629 (61.6)	221 (61.4)	408 (61.6)	0.939
Diabetes mellitus	285 (27.9)	99 (27.5)	186 (28.1)	0.839
Atrial fibrillation	410 (40.1)	155 (43.1)	255 (38.5)	0.158
Myocardial infarction	369 (36.1)	139 (38.6)	230 (34.7)	0.219
PCI	207 (20.3)7.0	61 (16.9)	146 (22.1)	0.052
CABG	149 (14.6)	59 (16.4)	90 (13.6)	0.227
Prior valve surgery	74 (7.2)	21 (5.8)	53 (8.0)	0.200
Peripheral artery disease	90 (8.8)	31 (8.6)	59 (8.9)	0.871
COPD	154 (15.1)	52 (14.4)	102 (15.4)	0.681
Stroke	93 (9.1)	33 (9.2)	60 (9.1)	0.956
Current malignancy	27 (2.6)	12 (3.3)	15 (2.3)	0.309
CKD	231 (22.6)	97 (26.9)	134 (20.2)	**0.014**
Device therapy				**0.018**
Pacemaker	67 (6.6)	30 (8.3)	37 (5.6)	
ICD	67 (6.6)	28 (7.8)	39 (5.9)	
CRT-P	19 (1.9)	8 (2.2)	11 (1.7)	
CRT-D	71 (7.0)	33 (9.2)	38 (5.7)	
Type of baseline visit				0.326
Inpatient hospitalization	617 (60.4)	210 (58.3)	407 (61.5)	
Outpatient clinic	405 (39.6)	150 (41.7)	255 (38.5)	
Reason for baseline visit				** < 0.001**
Worsening HF	444 (43.4)	185 (51.4)	259 (39.1)	
New-onset HF	311 (30.4)	76 (21.1)	235 (35.5)	
Other	267 (26.1)	99 (27.5)	168 (25.4)	
NYHA class				
Baseline				0.170
I	24 (2.4)	4 (1.1)	20 (3.1)	
II	454 (45.3)	156 (43.7)	298 (46.1)	
III	436 (43.5)	165 (46.2)	271 (42.0)	
IV	89 (8.9)	32 (9.0)	57 (8.8)	
9 months				** < 0.001**
I	190 (19.2)	38 (10.8)	152 (23.7)	
II	569 (57.4)	209 (59.5)	360 (56.2)	
III	219 (22.1)	97 (27.6)	122 (19.0)	
IV	14 (2.0)	7 (2.0)	7 (1.1)	
SBP				
Baseline	125 ± 22	123 ± 21	126 ± 22	**0.012**
9 months	124 ± 21	120 ± 20	127 ± 21	** < 0.001**
HF therapy				
ACEi/ARB				
Baseline use	802 (78.5)	278 (77.2)	524 (79.2)	0.473
3-month use	934 (91.4)	333 (92.5)	601 (90.8)	0.351
3-month target dose	270 (26.4)	71 (19.7)	199 (30.1)	** < 0.001**
3-month optimal dose fraction (%)	53 ± 40	48 ± 37	56 ± 42	**0.002**
β-Blockers				
Baseline use	867 (84.8)	310 (86.1)	557 (84.1)	0.401
3-month use	955 (93.4)	349 (96.9)	606 (91.5)	**0.001**
3-month target dose	141 (13.8)	49 (13.6)	92 (13.9)	0.899
3-month optimal dose fraction (%)	38 ± 30	37 ± 28	38 ± 31	0.881
MRA baseline use	562 (55.0)	216 (60.0)	346 (52.3)	**0.018**
Loop diuretic baseline use	1019 (99.7)	360 (100.0)	659 (99.6)	0.201
Digoxin baseline use	185 (18.1)	75 (20.8)	110 (16.6)	0.094

Data are presented as *n* (%) and mean ± standard deviation. Bold values represent significant* p*-values (*p* < 0.05)

*ACEi* angiotensin-converting enzyme inhibitor; *ARB* angiotensin receptor blocker; *BMI* body mass index; *CABG* coronary artery bypass graft; *CKD* chronic kidney disease; *COPD* chronic obstructive pulmonary disease; *CRT-D* cardiac resynchronization therapy with defibrillator; *CRT-P* cardiac resynchronization therapy with pacemaker; *HF* heart failure; *ICD* implantable cardioverter-defibrillator; *MR* mitral regurgitation; *MRA* mineralocorticoid receptor antagonist; *NYHA* New York Heart Association; *PCI* percutaneous coronary intervention; *SBP* systolic blood pressure

Median LVEF at 9 months was lower in patients with 9-month moderate-severe MR compared to those with no or mild MR (Table 2). Hence, patients with 9 month moderate-severe MR were more likely to have HFrEF at 9 months (LVEF < 40%) rather than HF with mid-range (HFmrEF; LVEF 40–49%) or preserved LVEF (HFpEF; LVEF ≥ 50%). Moreover, median LV end-diastolic diameter, LV end-systolic diameter, and left atrium diameter at baseline and at 9 months were all higher in patients with as compared to those without 9-month moderate-severe MR. Patients with 9-month moderate-severe MR had lower baseline eGFR and higher baseline and 9-month plasma NT-proBNP levels compared to patients with no or mild MR. All QoL measures assessed at 9 months were lower in these patients (Table 2).

**Table 2:** Echocardiographic data, laboratory data, and QoL measures in patients with vs. without 9-month moderate-severe MR

	Overall (*n* = 1022)	Moderate or severe MR (*n* = 360)	No or mild MR (*n* = 622)	*p* value
*Echocardiographic data*				
LVEF (%)				
Baseline	30 (25–35)	30 (25–35)	30 (25–35)	0.060
9 months	35 (28–42)	30 (25–38)	36 (30–45)	** < 0.001**
LVEF categories				
Baseline				0.409
HFrEF (LVEF < 40%)	810 (85.1)	298 (86.9)	512 (84.1)	
HFmrEF (LVEF 40–49%)	102 (10.7)	34 (9.9)	68 (11.2)	
HFpEF (LVEF ≥ 50%)	40 (4.2)	11 (3.2)	29 (4.8)	
9 months				** < 0.001**
HFrEF (LVEF < 40%)	611 (64.2)	269 (78.0)	342 (56.3)	
HFmrEF (LVEF 40–49%)	227 (23.8)	60 (17.4)	167 (27.5)	
HFpEF (LVEF ≥ 50%)	114 (12.0)	16 (4.6)	98 (16.1)	
LVEDD (mm)				
Baseline	62 (57–68)	64 (58–70)	61 (56–66)	** < 0.001**
9 months	61 (55–67)	64 (58–70)	60 (54–65)	** < 0.001**
LVESD (mm)				
Baseline	50 (44–56)	52 (46–59)	49 (43–55)	** < 0.001**
9 months	48 (40–56)	52 (45–60)	46 (39–52)	** < 0.001**
Left atrium diameter (mm)				
Baseline	47 (42–52)	49 (44–54)	46 (41–50)	** < 0.001**
9 months	46 (41–51)	48 (44–53)	44 (40–50)	** < 0.001**
*Laboratory data*				
Creatinine (µmol/L)				
Baseline	99 (81–124)	102 (83–127)	97 (80–123)	0.172
9 months	104 (84–131)	103 (86–135)	105 (83–130)	0.552
eGFR CKD-EPI (mL/min/1.73 m^2^)				
Baseline	64 (47–81)	61 (44–79)	65 (49–83)	**0.014**
9 months	60 (43–78)	60 (43–75)	60 (44–80)	0.214
Urea (mmol/L)				
Baseline	10.1 (7.1–16.1)	10.7 (7.4–17.1)	9.8 (7.0–15.6)	0.077
9 months	10.1 (7.0–16.1)	10.1 (7.2–17.9)	10.1 (6.8–15.4)	0.200
Sodium (mmol/L)				
Baseline	140 (137–142)	140 (138–142)	140 (137–142)	0.625
9 months	140 (137–142)	139 (137–142)	140 (138–142)	0.578
NT-proBNP (ng/L)				
Baseline	2056 (943–4785)	2659 (1206–5175)	1877 (859–4548)	** < 0.001**
9 months	1098 (371–2410)	1645 (765–3350)	781 (282–1889)	** < 0.001**
*QoL measures*				
6MWT distance (m)				
Baseline	282 (65–385)	268 (100–361)	294 (48–391)	0.240
9 months	350 (220–450)	314 (200–418)	360 (234–463)	** < 0.001**
KCCQ clinical summary score				
Baseline	54 (35–73)	51 (33–70)	56 (35–74)	0.123
9 months	70 (50–87)	63 (46–82)	73 (54–89)	** < 0.001**
KCCQ overall summary score				
Baseline	54 (36–72)	52 (36–70)	55 (38–73)	0.118
9 months	70 (51–85)	63 (47–80)	72 (55–88)	** < 0.001**
EQ-5D index value				
Baseline	0.74 (0.57–0.84)	0.74 (0.57–0.84)	0.74 (0.64–0.84)	0.597
9 months	0.78 (0.65–0.90)	0.77 (0.65–0.86)	0.81 (0.68–0.90)	** < 0.001**
EQ-5D VAS				
Baseline	60 (45–70)	51 (40–70)	60 (45–70)	**0.014**
9 months	65 (50–80)	60 (49–75)	70 (50–80)	** < 0.001**

Data are presented as *n* (%) and median (Q25–Q75). Bold values represent significant* p*-values (*p* < 0.05)

*6MWT* 6 min walking test; *CKD-EPI* chronic kidney disease epidemiology collaboration; *eGFR* estimated glomerular filtration rate; *EQ-5D* EuroQol-5 dimension; *HFmrEF* heart failure with mid-range ejection fraction; *HFpEF* heart failure with preserved ejection fraction; *HFrEF* heart failure with reduced ejection fraction; *KCCQ* Kansas city cardiomyopathy questionnaire; *LVEDD* left ventricular end-diastolic diameter; *LVEF* left ventricular ejection fraction; *LVESD* left ventricular end-systolic diameter; *MR* mitral regurgitation; *NT-proBNP* N-terminal pro-B-type natriuretic peptide; *QoL* quality-of-life; *VAS* visual analogue scale

### Impact of changes in MR severity and 9-month moderate-severe MR on clinical outcomes

**Fig. 1:**
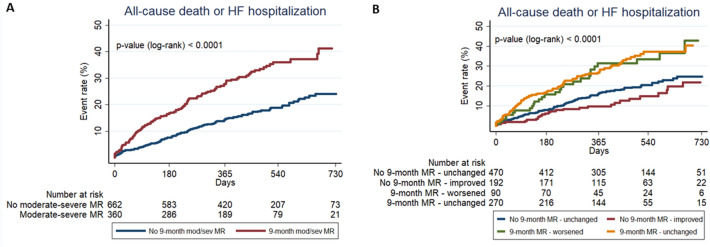
Primary endpoint. The figure shows Kaplan–Meier curves for 2-year primary endpoint (all-cause mortality or HF hospitalization) in patients with vs. without 9-month moderate-severe MR (panel A) and in four patients’ groups according to baseline and 9-month moderate-severe MR after GRMT optimization (panel B). *GRMT* guideline-directed medical therapy; *HF* heart failure; *MR* mitral regurgitation

After a median follow-up of 405 (IQR 254–554) days, the primary endpoint occurred in 114 patients (31.7%) with, and in 119 patients (18.0%) without, moderate-severe MR at 9 months (unadjusted HR 2.03, 95% CI 1.57–2.63). By Kaplan–Meier analysis, the incidence of the 2-year primary endpoint was higher in patients with, as compared to those without, moderate-severe MR at 9 months (log-rank *p* < 0.0001; Fig. 1A). Patients with worsened MR from baseline to 9 months and those with persistent moderate-severe MR at both baseline and 9 months had a similar incidence of the 2-year primary endpoint, which was higher compared to patients whose MR improved from baseline to 9 months and to those who had no or mild MR at both time-points (log-rank *p* < 0.0001; Fig. 1B). Hence, both patients with worsening MR and those with persistent moderate-severe MR at 9 months, after attempted GRMT, had a greater risk of the primary endpoint (unadjusted HR for worsening MR 1.78, 95% CI 1.17–2.72; unadjusted HR for persistent significant MR 1.91, 95% CI 1.42–2.57). Kaplan–Meier curves for all 2 year individual secondary endpoints are shown in Supplementary Figs. 2, 3, and 4. As shown in Supplementary Fig. 5, the higher risk of the primary endpoint in patients with 9-month moderate-severe MR was observed both in patients with LVEF < 40% and in those with LVEF ≥ 40% (*p* value for interaction = 0.803).

As shown in Table 3, 9-month moderate-severe MR was significantly associated with an increased risk of the primary endpoint, all-cause death, CV death, and HF hospitalization. The significant impact of 9 month moderate-severe MR on the primary endpoint was confirmed after multivariable adjustment for different models including age and sex (adjusted HR 1.83; 95% CI 1.42–2.38; *p* < 0.001); primary ischemic HF aetiology, baseline NYHA class, and previous HF hospitalization in last year (adjusted HR 1.62; 95% CI 1.24–2.11; *p* < 0.001); baseline LVEF categories, baseline eGFR, ACEi/ARB and β-blocker optimal dose fractions achieved at 3 months (adjusted HR 1.91; 95% CI 1.44–2.52; *p* < 0.001); 9 month LVEF categories, 9 month eGFR, ACEi/ARB and β-blocker optimal dose fractions achieved at 3 months (adjusted HR 1.68; 95% CI 1.23–2.29; *p* = 0.001); and the previously validated BIOSTAT-CHF risk prediction models (adjusted HR 1.85; 95% CI 1.43–2.39; *p* < 0.001). Similarly, the significant association between 9-month moderate–severe MR and all secondary endpoints was confirmed after adjustment for the same models.

**Table 3:** Cox regression models for the impact of 9-month moderate-severe MR on the combined endpoint (all-cause death or HF hospitalization), all-cause death, CV death and HF hospitalization

	Combined endpoint		All-cause death		CV death		HF hospitalization	
	HR (95% CI)	*p* value	HR (95% CI)	*p* value	HR (95% CI)	*p* value	HR (95% CI)	*p* value
Univariable analysis	2.03 (1.57–2.63)	** < 0.001**	1.83 (1.31–2.55)	** < 0.001**	1.86 (1.25–2.76)	**0.002**	2.09 (1.51–2.89)	** < 0.001**
Multivariable model 1 (adjusted for age and sex)	1.83 (1.42–2.38)	** < 0.001**	1.65 (1.18–2.30)	**0.003**	1.67 (1.12–2.48)	**0.012**	1.89 (1.36–2.62)	** < 0.001**
Multivariable model 2 (adjusted for primary ischemic HF aetiology, baseline NYHA class, and previous HF hospitalization in last year)	1.62 (1.24–2.11)	** < 0.001**	1.71 (1.22–2.39)	**0.002**	1.71 (1.15–2.55)	**0.009**	1.90 (1.37–2.65)	** < 0.001**
Multivariable model 3 (adjusted for baseline LVEF categories, baseline eGFR, ACEi/ARB optimal dose fraction at 3 months, and β-blocker optimal dose fraction at 3 months)	1.91 (1.44–2.52)	** < 0.001**	1.83 (1.27–2.64)	**0.001**	1.96 (1.27–3.03)	**0.002**	2.00 (1.41–2.84)	** < 0.001**
Multivariable model 4 (adjusted for LVEF categories at 9 months, eGFR at 9 months, ACEi/ARB optimal dose fraction at 3 months, and β-blocker optimal dose fraction at 3 months)	1.68 (1.23–2.29)	**0.001**	1.66 (1.11–2.47)	**0.013**	1.85 (1.15–2.99)	**0.011**	1.61 (1.09–2.39)	**0.017**
Multivariable model 5 (adjusted for BIOSTAT-CHF risk prediction models)*	1.85 (1.43–2.39)	** < 0.001**	1.74 (1.25–2.43)	**0.001**	1.76 (1.19–2.61)	**0.005**	1.85 (1.34–2.57)	** < 0.001**

Data are presented as HR and 95% CI. Bold values represent significant* p*-values (*p* < 0.05)

*ACEi* angiotensin-converting enzyme inhibitor; *ARB* angiotensin receptor blocker; *CI* confidence interval; *CV* cardiovascular; *eGFR* estimated glomerular filtration rate; *HF* heart failure; *HR* hazard ratio; *MR* mitral regurgitation; *LVEF* left ventricular ejection fraction; *NYHA* New York Heart Association; *NT-proBNP* N-terminal pro-B-type natriuretic peptide

^*^In multivariable model 5, 9-month moderate-to-severe MR was adjusted for the BIOSTAT-CHF risk prediction models, including the following covariates: age, HF hospitalization in last year, systolic blood pressure, peripheral oedema, log-NT-proBNP, haemoglobin, sodium, high-density lipoprotein, and use of β-blockers at baseline for the combined endpoint; age, log-urea, log-NT-proBNP, haemoglobin, and use of β-blockers at baseline for all-cause death and CV death; age, HF hospitalization in last year, systolic blood pressure, peripheral oedema, and estimated glomerular filtration rate for HF hospitalization

### Predictors of 9-month significant MR

At multivariable binary logistic regression analysis (Table 4), the presence of moderate-severe MR at baseline and older age were associated with an increased risk of 9 month moderate-severe MR, whereas HFpEF (LVEF ≥ 50% at baseline) and treatment with a higher fraction of ACEi/ARB optimal dose at 3 months were associated with a lower risk of 9 month moderate-severe MR. The *C* statistic (0.77) and Hosmer–Lemeshow goodness-of-fit test *p* value (0.48) confirmed good discrimination and fit of the multivariable model.

**Table 4:** Binary logistic regression analysis for the predictors of 9-month moderate-severe MR

	Univariable analysis		Multivariable analysis	
	OR (95% CI)	*p* value	OR (95% CI)	*p* value
Age (years)	1.02 (1.01–1.04)	** < 0.001**	1.03 (1.02–1.05)	** < 0.001**
Sex (women)	1.10 (0.81–1.48)	0.548	1.08 (0.72–1.60)	0.713
Primary ischemic HF aetiology	1.24 (0.96–1.61)	0.106	1.35 (0.96–1.89)	0.085
Previous HF hospitalization in last year	1.29 (0.97–1.71)	0.081	1.18 (0.83–1.70)	0.357
NYHA class III or IV	1.19 (0.92–1.55	0.181	1.00 (0.72–1.40)	0.994
Baseline moderate-severe MR	7.34 (5.49–9.83)	** < 0.001**	6.96 (4.96–9.78)	** < 0.001**
eGFR CKD-EPI (mL/min/1.73 m^2^)	0.99 (0.98–1.00)	**0.017**	1.00 (1.00–1.01)	0.273
Log-NT-proBNP (ng/L)	1.26 (1.12–1.40)	** < 0.001**	1.10 (0.94–1.27)	0.263
LVEF categories				
HFrEF (LVEF < 40%)—reference	**–**	**–**	**–**	–
HFmrEF (LVEF 40–49%)	0.86 (0.56–1.33)	0.494	0.82 (0.47–1.43)	0.491
HFpEF (LVEF ≥ 50%)	0.65 (0.32–1.32)	0.236	0.36 (0.16–0.85)	**0.019**
ACEi/ARB optimal dose fraction at 3 months (%)	0.59 (0.43–0.83)	**0.002**	0.60 (0.39–0.93)	**0.021**
β-Blocker optimal dose fraction at 3 months (%)	0.97 (0.63–1.49)	0.881	0.98 (0.57–1.70)	0.950

Data are presented as OR and 95% CI. Bold values represent significant *p*-values (*p* < 0.05). The *C *statistic for the multivariable model is 0.77, the Hosmer–Lemeshow goodness-of-fit test *p* value is 0.48

*ACEi* angiotensin-converting enzyme inhibitor; *ARB* angiotensin receptor blocker; *CI* confidence interval; *CKD-EPI* chronic kidney disease epidemiology collaboration; *eGFR* estimated glomerular filtration rate; *HF* heart failure; *HFmrEF* heart failure with mid-range ejection fraction; *HFpEF* heart failure with preserved ejection fraction; *HFrEF* heart failure with reduced ejection fraction; *LVEF* left ventricular ejection fraction; *MR* mitral regurgitation; *NYHA* New York Heart Association; *NT-proBNP* N-terminal pro-B-type natriuretic peptide; *OR* odds ratio

Regarding the prediction of changes in MR severity, among the 462 patients with moderate-severe MR at baseline, younger age, HFpEF, and treatment with a higher fraction of ACEi/ARB optimal dose at 3 months were associated with a lower likelihood of persistence of moderate-severe MR at 9 months (Supplementary Table 4). Among the 560 patients with no or mild MR at baseline, older age was the only independent predictor of worsening MR at 9 months (Supplementary Table 5).

## Discussion

The main findings of our study on patients with worsening or new-onset HF are as follows: (1) MR may dynamically change after attempted implementation of GRMT, with an improvement observed in a consistent proportion of patients with moderate-severe MR at baseline (41.6%), and MR development or worsening observed in a lower proportion of patients with no or mild MR at baseline (16.1%); (2) moderate-severe MR persists or develops despite GRMT in a substantial proportion of patients (35.2%) and has a strong prognostic impact even after adjusting for several other variables related to HF severity; (3) older age and presence of significant MR at baseline were associated with a greater risk of moderate-severe MR after GRMT optimization, whereas HFpEF and treatment with higher doses of ACEi/ARB were associated with a lower likelihood of moderate-severe MR after GRMT.

In our study, prevalence of 9 month moderate-severe MR after GRMT optimization in patients with HF was 35.2%, a figure that seems in line with prior studies not strictly focusing on GRMT optimization and reporting rates of moderate-severe MR ranging from 29 to 53% [1–3, 10, 13, 15, 16]. Furthermore, we found that MR severity was unchanged after GRMT optimization in 46.0% of patients without moderate-severe MR and in 26.4% of patients with moderate-severe MR, whereas MR improved in 18.8% and worsened in 8.8% of patients from baseline to 9 months. These results are in line with previous smaller studies reporting the evolution of MR in HFrEF patients receiving GRMT [37–40]. Compared to these studies [37–40], our analysis was performed on a much larger study group, including a broader HF population with HFrEF, HFmrEF and HFpEF patients. Despite the percentages of patients with improving or worsening MR were slightly lower in our study (18.8% and 8.8%, respectively) as compared to those reported by Nasser et al. (38% and 18%, respectively), both studies confirmed the prognostic impact of both persistent significant or worsening MR after GRMT optimization [38]. Of note, the Pharmacological Reduction of Functional, Ischemic Mitral Regurgitation (PRIME) trial randomized 118 patients with HF, LVEF < 50%, significant functional MR and optimized medical therapy with ACEi/ARBs or β-blockers to ARNI or valsartan, demonstrating a greater reduction in MR severity with ARNI at 1-year follow-up [28]. Although the benefits of ARNI could exceed those associated with ACEi/ARBs or β-blockers in terms of functional MR reduction, our study was performed before the introduction of ARNI into the routine clinical management of HF patients and, therefore, we could not evaluate the impact of this therapy. Several reasons could explain a lack of MR improvement or even MR worsening in a relevant proportion of patients (35.2%) in our study, including a more advanced stage of HF in terms of symptoms, clinical profile and echocardiographic findings, and lower odds of achieving higher GRMT doses during the optimization phase (see Supplementary Tables 1–2 for details). Biological variables, such as kidney dysfunction, hyperkalemia and low blood pressure may, on the other hand, cause lack of GRMT initiation or up-titration [41–43].

Our study demonstrates that both persistent significant MR and worsening MR after GRMT optimization in patients with HF are associated with an increased risk of all-cause death or HF hospitalization. The prognostic impact of moderate-severe MR after GRMT was particularly strong (univariable HR 2.03, 95% CI 1.57–2.63) and was confirmed after adjustment for several clinical, laboratory and echocardiographic variables, including the already validated BIOSTAT-CHF risk prediction model (adjusted HR 1.85, 95% CI 1.43–2.39) [33]. Our findings are in line with previous smaller studies showing the prognostic impact of persistent significant MR or worsening MR despite GRMT in HFrEF [38, 39]. Furthermore, a recent sub-analysis of the COAPT trial reported that MR improvement at 30 days was associated with better clinical outcomes and improved quality of life among HF patients, regardless of whether such improvement was achieved through transcatheter mitral valve repair or GRMT [44].

Since GRMT represents the first crucial therapeutic step in patients with HF and significant MR, [20, 21] our study may have clinically relevant implications in the management of patients with MR. Our data suggest that the optimal timing to evaluate interventional procedures for MR correction in HF should be after an adequate period of GRMT optimization since the prognostic impact of persistent significant MR after such period is particularly strong. In line with this concept, the COAPT trial demonstrated the prognostic benefit of percutaneous mitral valve repair in a carefully selected population of symptomatic patients with HF and persistent MR despite GRMT up-titration to maximally tolerated doses, as evaluated by a dedicated central committee [19, 24, 25]. Our study seems in line with this strategy of GRMT optimization before planning potential interventions for MR correction in HF.

Interestingly, ACEi/ARB up-titration was associated with a lower likelihood of persistence of moderate-severe MR in our study and, therefore, with MR improvement after the optimization phase. Prior randomized studies have already demonstrated that ACEi are effective in reducing functional MR in HFrEF [29]. Similarly, as already mentioned, sacubitril/valsartan was more effective than valsartan in reducing functional MR among 118 patients with HFrEF or HFmrEF enrolled in the PRIME trial [28]. Furthermore, among patients undergoing percutaneous mitral valve repair for secondary MR, in addition to a “COAPT-like” echocardiographic and clinical profile, GRMT was found to be a powerful predictor of 5 year survival [45]. Several mechanisms could explain the benefit of RAAS inhibitors on MR in HF, including reverse LV remodelling, afterload reduction and beneficial effects on valve leaflet remodelling [46, 47]. However, from the present study, we cannot assess whether the association between better up-titration of ACEi/ARB and a lower likelihood of persistence of moderate-severe MR was causally related.

Furthermore, we found that HFpEF was associated with a lower likelihood of moderate-severe MR after the optimization phase of GRMT up-titration. The different mechanisms and pathogenesis of MR in patients with normal or reduced LVEF could play a role in the different MR evolution in patients with HFrEF and HFpEF after GRMT optimization [48, 49]. Of note, atrial functional MR, typically associated with HFpEF and atrial fibrillation, has emerged as a distinct entity from “ventricular” secondary MR, typically associated with HFrEF [50]. Despite the better prognosis of patients with HFpEF as compared to those with HFrEF [51], significant functional MR has been associated with worse clinical outcomes both in HFpEF and in HFrEF patients [12]. However, the interplay between ACEi/ARB or β-blockers up-titration and persistence of significant MR in HFpEF needs to be further explored in dedicated studies.

### Limitations

Our study is a post hoc retrospective analysis of a prospective multicentre registry and, therefore, has all the usual limitations associated with this design. The main limitation is the lack of core-laboratory analysis of echocardiographic data and the consequent lack of detailed information regarding MR severity and aetiology. Furthermore, no independent adjudication of clinical events was performed. Of note, BIOSTAT-CHF was performed before the introduction of sodium-glucose cotransporter-2 inhibitors and ARNI in the treatment of HFrEF, hence the impact of these therapies on MR evolution and prognosis could not be evaluated. Moreover, the proportion of patients with HFpEF in our study was relatively small (4.2%), thus preventing from definitive conclusions for this disease entity, and no specific recommendations regarding GRMT were available for HFpEF at time of BIOSTAT-CHF enrolment [35].

## Conclusions

In patients with worsening or new-onset HF enrolled in BIOSTAT-CHF, MR severity may dynamically change after a dedicated period of GRMT optimization. The presence of moderate-severe MR after GRMT optimization, either persistent moderate-severe or worsening MR from baseline, has a strong prognostic impact, regardless of relevant variables of interest and from an already validated risk prediction model. Higher ACEi/ARB up-titration and HFpEF were associated with a higher likelihood of MR improvement after GRMT optimization.

## Supplementary Information

Below is the link to the electronic supplementary material.

Supplementary file1 (DOCX 8597 KB)
