# Supplementary material for: Clinical impact of changes in mitral regurgitation severity after medical therapy optimization in heart failure
Source: Clin Res Cardiol. 2022 Mar 16;111(8):912–23. doi: 10.1007/s00392-022-01991-7 (PMC9334376; doi:10.1007/s00392-022-01991-7)
Supplement: Supplementary file 1 — Supplementary file1 (DOCX 8597 KB) [file 392_2022_1991_MOESM1_ESM.docx]

**SUPPLEMENTARY APPENDIX**

**Supplementary Table 1 – Clinical characteristics according to baseline and 9-month moderate-severe MR**

|  | **No or Mild MR at Baseline and 9-month – Unchanged**  **(n=470)** | **Moderate or Severe MR at Baseline, No or Mild MR at 9-month – Improved**  **(n=192)** | **No or Mild MR at Baseline, Moderate or Severe MR at 9-month – Worsened (n=90)** | **Moderate or Severe MR at Baseline and 9-month – Unchanged (n=270)** | ***p*-value** |
| --- | --- | --- | --- | --- | --- |
| Age (years) | 66.1 ± 12.7 | 64.8 ± 12.1 | 71.4 ± 10.0 | 68.4 ± 11.4 | **<0.001** |
| Men | 371 (78.9) | 142 (74.0) | 65 (72.2) | 208 (77.0) | 0.370 |
| BMI (kg/m^2^) | 28.7 ± 5.8 | 27.2 ± 5.4 | 26.3 ± 4.8 | 27.2 ± 4.6 | **<0.001** |
| HF hospitalization in last year | 121 (25.7) | 51 (26.6) | 29 (32.2) | 83 (30.7) | 0.365 |
| Primary ischemic HF etiology | 207 (44.6) | 67 (35.6) | 47 (52.2) | 121 (45.7) | **0.043** |
| Medical history (baseline) |  |  |  |  |  |
| Hypertension | 299 (63.6) | 109 (56.8) | 53 (58.9) | 168 (62.2) | 0.388 |
| Diabetes mellitus | 139 (29.6) | 47 (24.5) | 23 (25.6) | 76 (28.2) | 0.567 |
| Atrial fibrillation | 184 (39.2) | 71 (37.0) | 36 (40.0) | 119 (44.1) | 0.435 |
| Myocardial infarction | 170 (36.2) | 60 (31.3) | 43 (47.8) | 96 (35.6) | 0.063 |
| PCI | 108 (23.0) | 38 (19.8) | 23 (25.6) | 38 (14.1) | **0.017** |
| CABG | 71 (15.1) | 19 (9.9) | 19 (21.1) | 40 (14.8) | 0.086 |
| Prior valve surgery | 39 (8.3) | 14 (7.3) | 4 (4.4) | 17 (6.3) | 0.534 |
| Peripheral artery disease | 43 (9.2) | 16 (8.3) | 4 (4.4) | 27 (10.0) | 0.435 |
| COPD | 76 (16.2) | 26 (13.5) | 14 (15.6) | 38 (14.1) | 0.796 |
| Stroke | 44 (9.4) | 16 (8.3) | 6 (6.7) | 27 (10.0) | 0.781 |
| Current malignancy | 13 (2.8) | 2 (1.0) | 5 (5.6) | 7 (2.6) | 0.178 |
| CKD | 95 (20.2) | 39 (20.3) | 24 (26.7) | 73 (27.0) | 0.112 |
| Device therapy |  |  |  |  | 0.055 |
| Pacemaker | 31 (6.6) | 6 (3.1) | 6 (6.7) | 24 (8.9) |  |
| ICD | 32 (6.8) | 7 (3.7) | 8 (8.9) | 20 (7.4) |  |
| CRT-P | 6 (1.3) | 5 (2.6) | 1 (1.1) | 7 (2.6) |  |
| CRT-D | 27 (5.7) | 11 (5.7) | 8 (8.9) | 25 (9.3) |  |
| Type of baseline visit |  |  |  |  | 0.667 |
| Inpatient hospitalization | 284 (60.4) | 123 (64.1) | 51 (56.7) | 159 (58.9) |  |
| Outpatient clinic | 186 (39.6) | 69 (35.9) | 39 (43.3) | 111 (41.1) |  |
| Reason for baseline visit |  |  |  |  | **<0.001** |
| Worsening HF | 190 (40.4) | 69 (35.9) | 44 (48.9) | 141 (52.2) |  |
| New-onset HF | 160 (34.0) | 75 (39.1) | 19 (21.1) | 57 (21.1) |  |
| Other | 120 (25.5) | 48 (25.0) | 27 (30.0) | 72 (26.7) |  |
| NYHA class |  |  |  |  |  |
| Baseline |  |  |  |  | 0.056 |
| I | 17 (3.7) | 3 (1.6) | 1 (1.1) | 3 (1.1) |  |
| II | 219 (47.6) | 79 (42.5) | 37 (41.6) | 119 (44.4) |  |
| III | 193 (42.0) | 78 (41.9) | 44 (49.4) | 121 (45.2) |  |
| IV | 31 (6.7) | 26 (14.0) | 7 (7.9) | 25 (9.3) |  |
| 9-month |  |  |  |  | **<0.001** |
| I | 95 (20.7) | 57 (31.3) | 10 (11.2) | 28 (10.7) |  |
| II | 269 (58.6) | 91 (50.0) | 49 (55.1) | 160 (61.1) |  |
| III | 89 (19.4) | 33 (18.1) | 28 (31.5) | 69 (26.3) |  |
| IV | 6 (1.3) | 1 (0.6) | 2 (2.3) | 5 (1.9) |  |
| SBP |  |  |  |  |  |
| Baseline | 127 ± 22 | 125 ± 21 | 125 ± 25 | 122 ± 19 | **0.028** |
| 9-month | 127 ± 22 | 127 ± 20 | 118 ± 20 | 121 ± 20 | **<0.001** |
| HF therapy |  |  |  |  |  |
| ACEi/ARB |  |  |  |  |  |
| Baseline use | 380 (80.9) | 144 (75.0) | 64 (71.1) | 214 (79.3) | 0.115 |
| 3-month use | 430 (91.5) | 171 (89.1) | 83 (92.2) | 250 (92.6) | 0.593 |
| 3-month target dose | 139 (29.6) | 60 (31.3) | 17 (18.9) | 54 (20.0) | **0.005** |
| 3-month optimal dose fraction (%) | 55 ± 41 | 57 ± 43 | 48 ± 39 | 48 ± 36 | **0.024** |
| β-blockers |  |  |  |  |  |
| Baseline use | 400 (85.1) | 157 (81.8) | 78 (86.7) | 232 (85.9) | 0.591 |
| 3-month use | 430 (91.5) | 176 (91.7) | 87 (96.7) | 262 (97.0) | **0.011** |
| 3-month target dose | 63 (13.4) | 29 (15.1) | 14 (15.6) | 35 (13.0) | 0.866 |
| 3-month optimal dose fraction (%) | 38 ± 31 | 38 ± 31 | 36 ± 30 | 38 ± 28 | 0.961 |
| MRA baseline use | 241 (51.3) | 105 (54.7) | 48 (53.3) | 168 (62.2) | **0.038** |
| Loop diuretic baseline use | 469 (99.8) | 190 (99.0) | 90 (100.0) | 270 (100.0) | 0.184 |
| Digoxin baseline use | 76 (16.2) | 34 (17.7) | 13 (14.4) | 62 (23.0) | 0.097 |
|  |  |  |  |  |  |

Data are presented as n (%) and mean ± standard deviation.

ACEi = angiotensin-converting enzyme inhibitor; ARB = angiotensin receptor blocker; BMI = body mass index; CABG = coronary artery bypass graft; CKD = chronic kidney disease; COPD = chronic obstructive pulmonary disease; CRT-D = cardiac resynchronization therapy with defibrillator; CRT-P = cardiac resynchronization therapy with pacemaker; HF = heart failure; ICD = implantable cardioverter-defibrillator; MR = mitral regurgitation; MRA = mineralocorticoid receptor antagonist; NYHA = New York Heart Association; PCI = percutaneous coronary intervention.

|  | **No or Mild MR at Baseline and 9-month – Unchanged**  **(n=470)** | **Moderate or Severe MR at Baseline, No or Mild MR at 9-month – Improved**  **(n=192)** | **No or Mild MR at Baseline, Moderate or Severe MR at 9-month – Worsened (n=90)** | **Moderate or Severe MR at Baseline and 9-month – Unchanged (n=270)** | ***p*-value** |
| --- | --- | --- | --- | --- | --- |
| ***Echocardiographic data*** |  |  |  |  |  |
| LVEF (%) |  |  |  |  |  |
| Baseline | 30 (25-35) | 28 (23-35) | 30 (25-36) | 30 (23-35) | **0.003** |
| 9-month | 36 (30-45) | 35 (30-45) | 30 (25-38) | 30 (25-38) | **<0.001** |
| LVEF categories |  |  |  |  |  |
| Baseline |  |  |  |  | 0.062 |
| HFrEF (LVEF <40%) | 356 (84.0) | 156 (84.3) | 68 (81.0) | 230 (88.8) |  |
| HFmrEF (LVEF 40-49%) | 52 (12.3) | 16 (8.7) | 14 (16.7) | 20 (7.7) |  |
| HFpEF (LVEF ≥50%) | 16 (3.8) | 13 (7.0) | 2 (2.4) | 9 (3.5) |  |
| 9-month |  |  |  |  | **<0.001** |
| HFrEF (LVEF <40%) | 236 (55.4) | 106 (58.6) | 67 (78.8) | 202 (77.7) |  |
| HFmrEF (LVEF 40-49%) | 123 (28.9) | 44 (24.3) | 16 (18.8) | 44 (16.9) |  |
| HFpEF (LVEF ≥50%) | 67 (15.7) | 31 (17.1) | 2 (2.4) | 14 (5.4) |  |
| LVEDD (mm) |  |  |  |  |  |
| Baseline | 61 (56-65) | 63 (58-68) | 61 (55-70) | 65 (59-70) | **<0.001** |
| 9-month | 59 (54-65) | 61 (54-65) | 64 (58-70) | 64 (58-70) | **<0.001** |
| LVESD (mm) |  |  |  |  |  |
| Baseline | 48 (43-54) | 52 (46-58) | 50 (42-56) | 53 (47-60) | **<0.001** |
| 9-month | 46 (39-52) | 47 (41-53) | 52 (44-61) | 52 (45-60) | **<0.001** |
| Left atrium diameter (mm) |  |  |  |  |  |
| Baseline | 45 (40-50) | 47 (43-52) | 45 (42-50) | 49 (45-55) | **<0.001** |
| 9-month | 44 (40-50) | 44 (40-49) | 47 (44-52) | 49 (45-54) | **<0.001** |
| ***Laboratory data*** |  |  |  |  |  |
| Creatinine (µmol/L) |  |  |  |  |  |
| Baseline | 97 (81-123) | 96 (79-123) | 97 (80-120) | 106 (84-129) | 0.263 |
| 9-month | 104 (81-129) | 106 (88-133) | 99 (81-122) | 104 (88-137) | 0.233 |
| eGFR CKD-EPI (mL/min/1.73 m^2^) |  |  |  |  |  |
| Baseline | 65 (49-83) | 64 (49-84) | 64 (47-77) | 60 (43-79) | 0.095 |
| 9-month | 60 (46-81) | 60 (43-78) | 61 (44-78) | 59 (42-75) | 0.280 |
| Urea (mmol/L) |  |  |  |  |  |
| Baseline | 9.8 (7.1-15.7) | 10.2 (7.0-15.4) | 10.5 (7.4-15.7 | 11.0 (7.4-18.2) | 0.352 |
| 9-month | 9.8 (6.7-15.5) | 10.9 (7.9-15.0) | 9.5 (7.0-18.1) | 10.3 (7.3-17.9) | 0.412 |
| Sodium (mmol/L) |  |  |  |  |  |
| Baseline | 140 (137-142) | 140 (138-142) | 140 (138-142) | 140 (137-142) | 0.771 |
| 9-month | 140 (138-142) | 140 (138-142) | 140 (138-142) | 139 (137-142) | 0.840 |
| NT-proBNP (ng/L) |  |  |  |  |  |
| Baseline | 1718 (739-4333) | 2374 (1087-4884) | 2554 (1064-5671) | 2778 (1276-5119) | **<0.001** |
| 9-month | 811 (289-1882) | 748 (247-1894) | 1515 (619-2914) | 1708 (807-3838) | **<0.001** |
| ***QoL measures*** |  |  |  |  |  |
| 6MWT distance (m) |  |  |  |  |  |
| Baseline | 288 (33-390) | 306 (67-400) | 258 (43-381) | 268 (120-360) | 0.473 |
| 9-month | 352 (218-465) | 390 (286-460) | 336 (206-434) | 312 (200-410) | **<0.001** |
| KCCQ clinical summary score |  |  |  |  |  |
| Baseline | 56 (36-75) | 54 (32-71) | 49 (36-70) | 51 (32-70) | 0.254 |
| 9-month | 71 (51-87) | 79 (57-92) | 61 (45-87) | 64 (46-81) | **<0.001** |
| KCCQ overall summary score |  |  |  |  |  |
| Baseline | 56 (39-73) | 53 (33-70) | 52 (38-69) | 52 (35-70) | 0.204 |
| 9-month | 71 (52-86) | 77 (59-90) | 63 (48-83) | 64 (47-79) | **<0.001** |
| EQ-5D index value |  |  |  |  |  |
| Baseline | 0.74 (0.62-0.84) | 0.77 (0.65-0.84) | 0.74 (0.57-0.86) | 0.74 (0.57-0.81) | 0.881 |
| 9-month | 0.78 (0.66-0.90) | 0.81 (0.69-1.00) | 0.74 (0.57-0.90) | 0.77 (0.65-0.86) | **0.002** |
| EQ-5D VAS |  |  |  |  |  |
| Baseline | 60 (45-70) | 60 (45-70) | 50 (40-70) | 55 (40-70) | 0.102 |
| 9-month | 65 (50-80) | 70 (55-80) | 60 (40-75) | 60 (50-77) | **<0.001** |
|  |  |  |  |  |  |

**Supplementary Table 2 – Echocardiographic data, laboratory characteristics, and QoL measures according to 9-month and baseline moderate-severe MR.**

Data are presented as n (%) and median (Q25-Q75).

6MWT = 6-minute walking test; CKD-EPI = Chronic Kidney Disease Epidemiology Collaboration; eGFR = estimated glomerular filtration rate; EQ-5D = EuroQol - 5 Dimension; HFmrEF = heart failure with mid-range ejection fraction; HFpEF = heart failure with preserved ejection fraction; HFrEF = heart failure with reduced ejection fraction; KCCQ = Kansas City Cardiomyopathy Questionnaire; LVEDD = left ventricular end-diastolic diameter; LVEF = left ventricular ejection fraction; LVESD = left ventricular end-systolic diameter; MR = mitral regurgitation; NT-proBNP = N-terminal pro-B-type natriuretic peptide; QoL = quality-of-life; VAS = Visual Analogue Scale.

**Supplementary Table 3 – Clinical characteristics among patients included vs. not included in the study.**

|  | **Included (n=1022)** | **Excluded**  **(n=1494)** | ***p*-value** |
| --- | --- | --- | --- |
| Age (years) | 66.9 ± 12.2 | 70.2 ± 11.6 | **<0.001** |
| Men | 786 (76.9) | 1060 (71.0) | **0.001** |
| BMI (kg/m^2^) | 27.8 ± 5.4 | 27.9 ± 5.6 | 0.688 |
| HF hospitalization in last year | 284 (27.8) | 510 (34.1) | **0.001** |
| Primary ischemic HF aetiology | 442 (43.9) | 684 (46.7) | 0.175 |
| Medical history |  |  |  |
| Hypertension | 629 (61.6) | 940 (62.9) | 0.485 |
| Diabetes mellitus | 285 (27.9) | 534 (35.7) | **0.001** |
| Atrial fibrillation | 410 (40.1) | 733 (49.1) | **<0.001** |
| Myocardial infarction | 369 (36.1) | 594 (39.8) | 0.064 |
| PCI | 207 (20.3) | 337 (22.6) | 0.168 |
| CABG | 149 (14.6) | 284 (19.0) | **0.004** |
| Prior valve surgery | 74 (7.2) | 105 (7.0) | 0.839 |
| Peripheral artery disease | 90 (8.8) | 183 (12.3) | **0.006** |
| COPD | 154 (15.1) | 282 (18.9) | **0.013** |
| Stroke | 93 (9.1) | 140 (9.4) | 0.818 |
| Current malignancy | 27 (2.6) | 70 (4.7) | **0.009** |
| CKD | 231 (22.6) | 465 (31.1) | **<0.001** |
| Device therapy |  |  | 0.151 |
| Pacemaker | 67 (6.6) | 116 (7.8) |  |
| ICD | 67 (6.6) | 138 (9.2) |  |
| CRT-P | 19 (1.9) | 30 (2.0) |  |
| CRT-D | 71 (7.0) | 102 (6.8) |  |
| Type of baseline visit |  |  | **<0.001** |
| Inpatient hospitalization | 617 (60.4) | 1077 (72.1) |  |
| Outpatient clinic | 405 (39.6) | 417 (27.9) |  |
| Reason for baseline visit |  |  | **<0.001** |
| Worsening HF | 444 (43.4) | 928 (62.1) |  |
| New-onset HF | 311 (30.4) | 391 (26.2) |  |
| Other | 267 (26.1) | 175 (11.7) |  |
| NYHA class |  |  |  |
| Baseline |  |  | **<0.001** |
| I | 24 (2.4) | 32 (2.2) |  |
| II | 454 (45.3) | 414 (28.7) |  |
| III | 436 (43.5) | 792 (54.9) |  |
| IV | 89 (8.9) | 205 (14.2) |  |
| 9-month |  |  | **0.001** |
| I | 190 (19.2) | 126 (13.6) |  |
| II | 569 (57.4) | 538 (58.1) |  |
| III | 219 (22.1) | 254 (27.4) |  |
| IV | 14 (1.4) | 8 (0.9) |  |
| SBP |  |  |  |
| Baseline | 125 ± 22 | 124 ± 22 | 0.528 |
| 9-month | 124 ± 21 | 125 ± 20 | 0.704 |
| HF therapy |  |  |  |
| ACEi/ARB |  |  |  |
| Baseline use | 802 (78.5) | 1018 (68.1) | **<0.001** |
| 3-month use | 934 (91.4) | 1259 (84.3) | **<0.001** |
| 3-month target dose | 270 (26.4) | 276 (18.5) | **<0.001** |
| 3-month optimal dose fraction (%) | 53 ± 40 | 44 ± 36 | **<0.001** |
| β-blockers |  |  |  |
| Baseline use | 867 (84.8) | 1226 (82.1) | 0.068 |
| 3-month use | 955 (93.4) | 1342 (89.8) | **0.002** |
| 3-month target dose | 141 (13.8) | 178 (11.9) | 0.163 |
| 3-month optimal dose fraction (%) | 38 ± 30 | 35 ± 31 | **0.015** |
| MRA baseline use | 562 (55.0) | 777 (52.0) | 0.141 |
| Loop diuretic baseline use | 1019 (99.7) | 1485 (99.4) | 0.269 |
| Digoxin baseline use | 185 (18.1) | 306 (20.5) | 0.139 |
|  |  |  |  |

Data are presented as n (%) and mean ± standard deviation.

ACEi = angiotensin-converting enzyme inhibitor; ARB = angiotensin receptor blocker; BMI = body mass index; CABG = coronary artery bypass graft; CKD = chronic kidney disease; COPD = chronic obstructive pulmonary disease; CRT-D = cardiac resynchronization therapy with defibrillator; CRT-P = cardiac resynchronization therapy with pacemaker; HF = heart failure; ICD = implantable cardioverter-defibrillator; MR = mitral regurgitation; MRA = mineralocorticoid receptor antagonist; NYHA = New York Heart Association; PCI = percutaneous coronary intervention; SBP = systolic blood pressure.

**Supplementary Table 4 – Multivariable binary logistic regression analysis for the predictors of persistent moderate-severe MR (among patients with moderate-severe MR at baseline).**

|  | **Univariable analysis** | | **Multivariable analysis** | |
| --- | --- | --- | --- | --- |
|  | **OR (95% CI)** | ***p*-value** | **OR (95% CI)** | ***p*-value** |
| Age (years) | 1.03 (1.01-1.04) | **0.001** | 1.02 (1.00-1.05) | **0.029** |
| Sex (women) | 0.85 (0.55-1.30) | 0.447 | 0.95 (0.57-1.60) | 0.857 |
| Primary ischemic HF etiology | 1.52 (1.03-2.23) | **0.033** | 1.53 (0.98-2.38) | 0.061 |
| Previous HF hospitalization in last year | 1.23 (0.81-1.85) | 0.330 | 0.84 (0.54-1.29) | 0.423 |
| NYHA class III or IV | 0.94 (0.65-1.37) | 0.762 | 0.98 (0.62-1.57) | 0.949 |
| eGFR CKD-EPI (mL/min/1.73 m^2^) | 0.99 (0.98-1.00) | 0.070 | 1.00 (0.99-1.01) | 0.733 |
| Log-NT-proBNP (ng/L) | 1.09 (0.92-1.29) | 0.324 | 1.02 (0.83-1.26) | 0.815 |
| LVEF categories |  |  |  |  |
| HFrEF (LVEF <40%) – reference | **-** | **-** | **-** | **-** |
| HFmrEF (LVEF 40-49%) | 0.85 (0.43-1.69) | 0.638 | 0.59 (0.27-1.27) | 0.178 |
| HFpEF (LVEF ≥50%) | 0.47 (0.20-1.13) | 0.090 | 0.37 (0.14-1.00) | **0.049** |
| ACEi/ARB optimal dose fraction at 3 months (%) | 0.56 (0.35-0.90) | **0.017** | 0.54 (0.31-0.94) | **0.030** |
| β-blocker optimal dose fraction at 3 months (%) | 1.00 (0.53-1.90) | 0.990 | 1.20 (0.58-2.49) | 0.619 |
|  |  |  |  |  |

Data are presented as OR and 95% CI. The *C-*statistic for the multivariable model is 0.63, the Hosmer-Lemeshow goodness-of-fit test p-value is 0.32.

ACEi = angiotensin-converting enzyme inhibitor; ARB = angiotensin receptor blocker; CI = confidence interval; CKD-EPI = Chronic Kidney Disease Epidemiology Collaboration; eGFR = estimated glomerular filtration rate; HF = heart failure; HFmrEF = heart failure with mid-range ejection fraction; HFpEF = heart failure with preserved ejection fraction; HFrEF = heart failure with reduced ejection fraction; LVEF = left ventricular ejection fraction; MR = mitral regurgitation; NYHA = New York Heart Association; NT-proBNP = N-terminal pro-B-type natriuretic peptide; OR = odds ratio.

**Supplementary Table 5 – Multivariable binary logistic regression analysis for the predictors of worsening MR (among patients with no or mild MR at baseline).**

|  | **Univariable analysis** | | **Multivariable analysis** | |
| --- | --- | --- | --- | --- |
|  | **OR (95% CI)** | ***p*-value** | **OR (95% CI)** | ***p*-value** |
| Age (years) | 1.04 (1.02-1.06) | **<0.001** | 1.05 (1.02-1.08) | **0.001** |
| Sex (women) | 1.44 (0.86-2.40) | 0.162 | 1.28 (0.69-2.36) | 0.433 |
| Primary ischemic HF etiology | 1.36 (0.86-2.13) | 0.186 | 1.17 (0.69-2.00) | 0.562 |
| Previous HF hospitalization in last year | 1.37 (0.84-2.23) | 0.205 | 1.48 (0.84-2.59) | 0.175 |
| NYHA class III or IV | 1.41 (0.89-2.24) | 0.138 | 1.22 (0.71-2.11) | 0.476 |
| eGFR CKD-EPI (mL/min/1.73 m^2^) | 1.00 (0.98-1.01) | 0.350 | 1.01 (0.99-1.02) | 0.255 |
| Log-NT-proBNP (ng/L) | 1.23 (1.02-1.49) | **0.030** | 1.14 (0.91-1.44) | 0.257 |
| LVEF categories |  |  |  |  |
| HFrEF (LVEF <40%) – reference | **-** | **-** | **-** | **-** |
| HFmrEF (LVEF 40-49%) | 1.41 (0.74-2.69) | 0.297 | 1.18 (0.56-2.48) | 0.672 |
| HFpEF (LVEF ≥50%) | 0.65 (0.15-2.91) | 0.578 | 0.42 (0.09-2.03) | 0.283 |
| ACEi/ARB optimal dose fraction at 3 months (%) | 0.61 (0.33-1.10) | 0.100 | 0.70 (0.34-1.43) | 0.327 |
| β-blocker optimal dose fraction at 3 months (%) | 0.84 (0.40-1.77) | 0.641 | 0.78 (0.33-1.87) | 0.580 |
|  |  |  |  |  |

Data are presented as OR and 95% CI. The *C-*statistic for the multivariable model is 0.68, the Hosmer-Lemeshow goodness-of-fit test p-value is 0.52.

ACEi = angiotensin-converting enzyme inhibitor; ARB = angiotensin receptor blocker; CI = confidence interval; CKD-EPI = Chronic Kidney Disease Epidemiology Collaboration; eGFR = estimated glomerular filtration rate; HF = heart failure; HFmrEF = heart failure with mid-range ejection fraction; HFpEF = heart failure with preserved ejection fraction; HFrEF = heart failure with reduced ejection fraction; LVEF = left ventricular ejection fraction; MR = mitral regurgitation; NYHA = New York Heart Association; NT-proBNP = N-terminal pro-B-type natriuretic peptide; OR = odds ratio.

**Supplementary Figure 1 – Study flow-chart.**


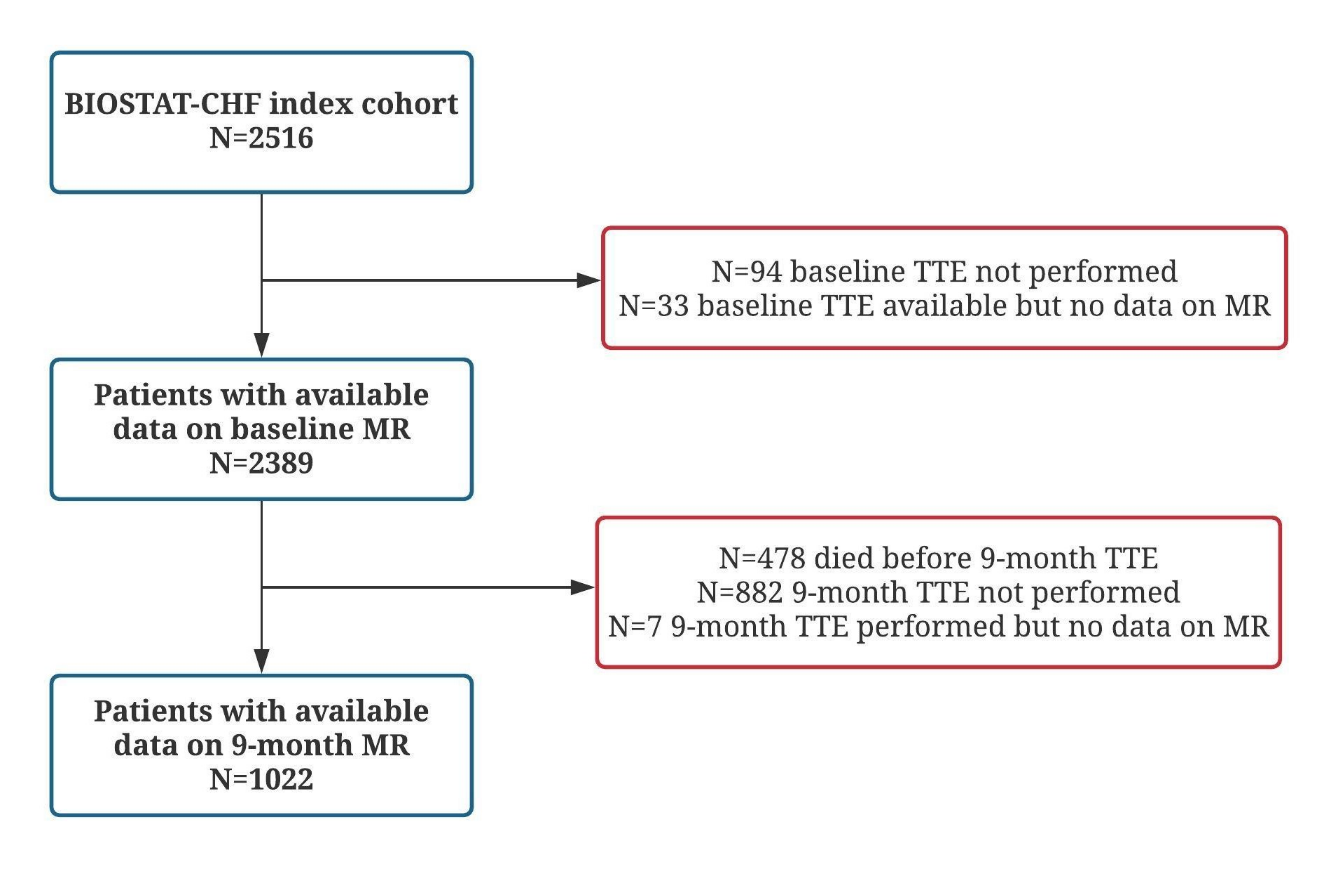


The figure shows the flow-chart of included patients from BIOSTAT-CHF index cohort.

MR = mitral regurgitation; TTE = transthoracic echocardiography.

**Supplementary Figure 2 – All-cause mortality.**

**
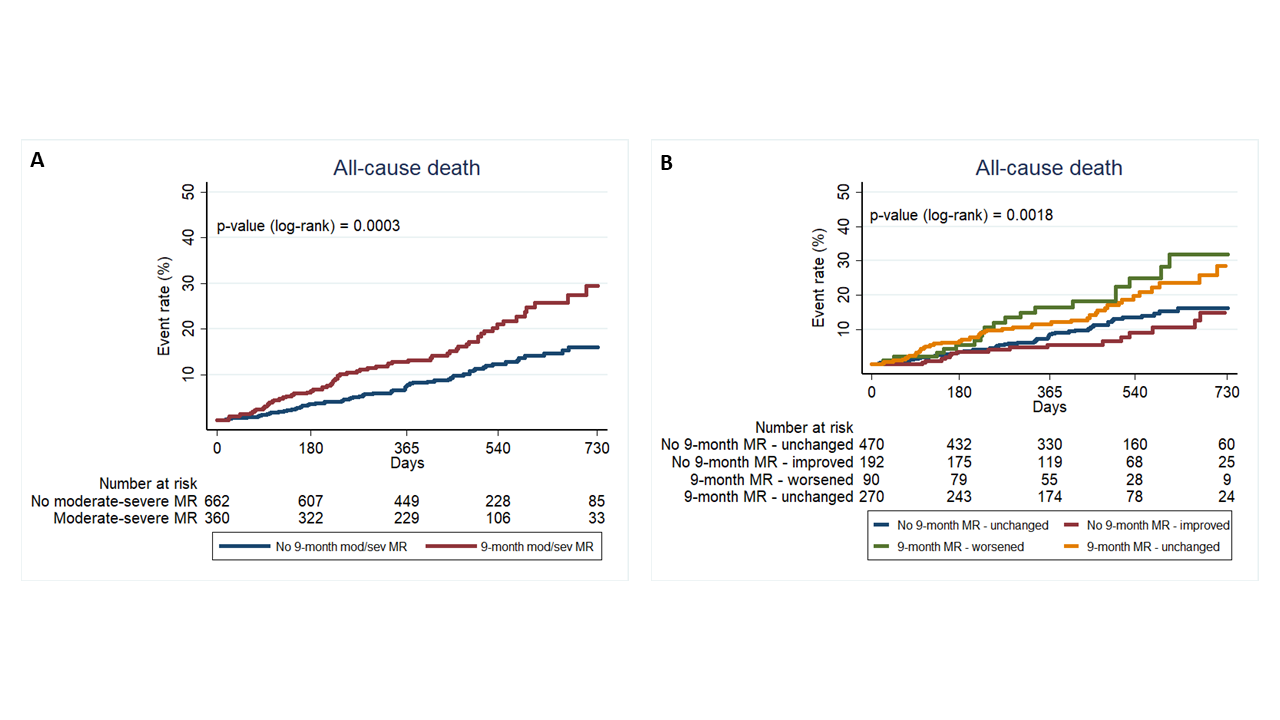
**

The figure shows Kaplan-Meier curves for 2-year all-cause mortality in patients with vs. without 9-month moderate-severe MR (panel A) and in four patients’ groups according to baseline and 9-month moderate-severe MR after GRMT optimization (panel B).

GRMT = guideline-directed medical therapy; MR = mitral regurgitation.

**Supplementary Figure 3 – Cardiovascular mortality.**

**
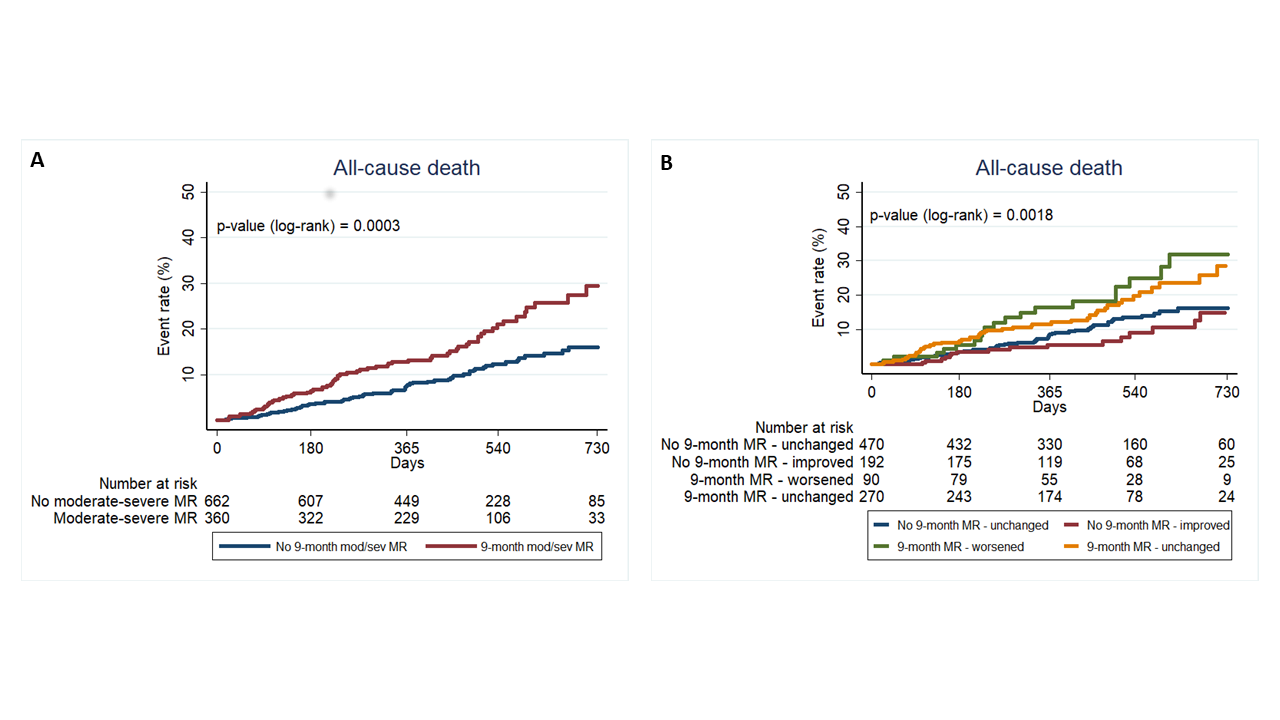
**

The figure shows Kaplan-Meier curves for 2-year cardiovascular mortality in patients with vs. without 9-month moderate-severe MR (panel A) and in four patients’ groups according to baseline and 9-month moderate-severe MR after GRMT optimization (panel B).

GRMT = guideline-directed medical therapy; MR = mitral regurgitation.

**Supplementary Figure 4 – HF hospitalization.**


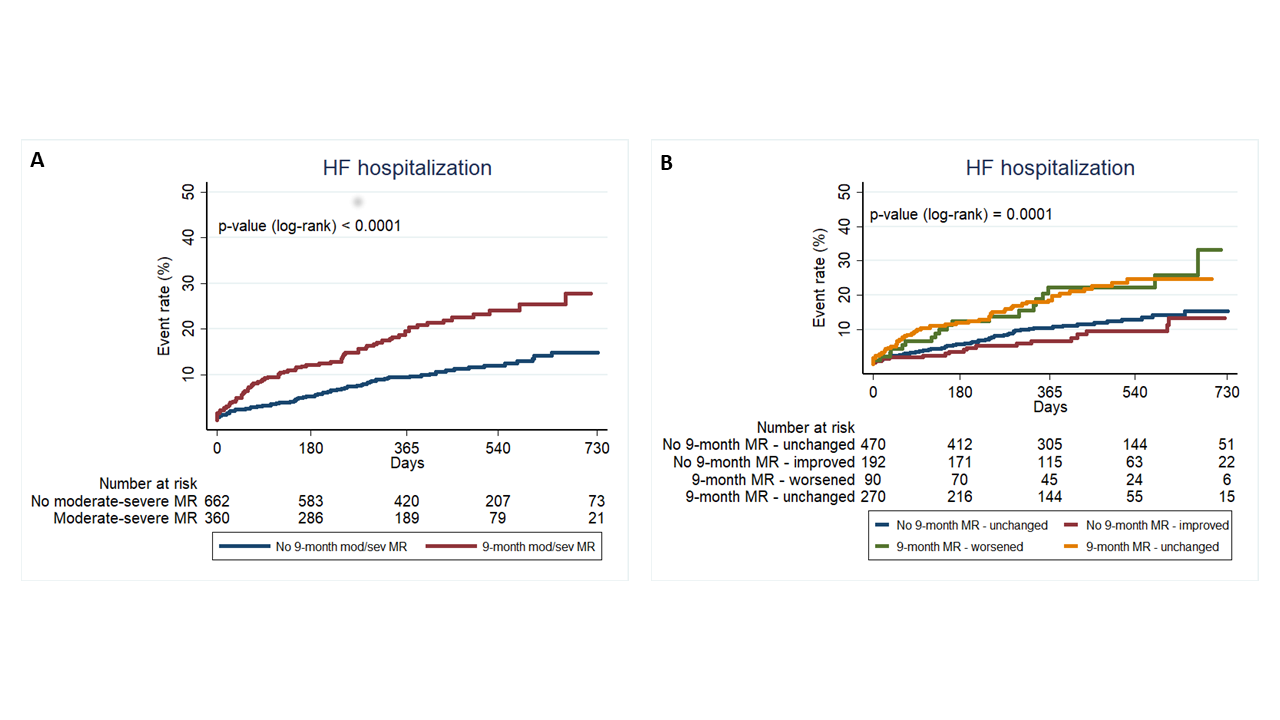


The figure shows Kaplan-Meier curves for 2-year HF hospitalization in patients with vs. without 9-month moderate-severe MR (panel A) and in four patients’ groups according to baseline and 9-month moderate-severe MR after GRMT optimization (panel B).

GRMT = guideline-directed medical therapy; HF = heart failure; MR = mitral regurgitation.

**Supplementary Figure 5 – Primary endpoint in LVEF subgroups.**


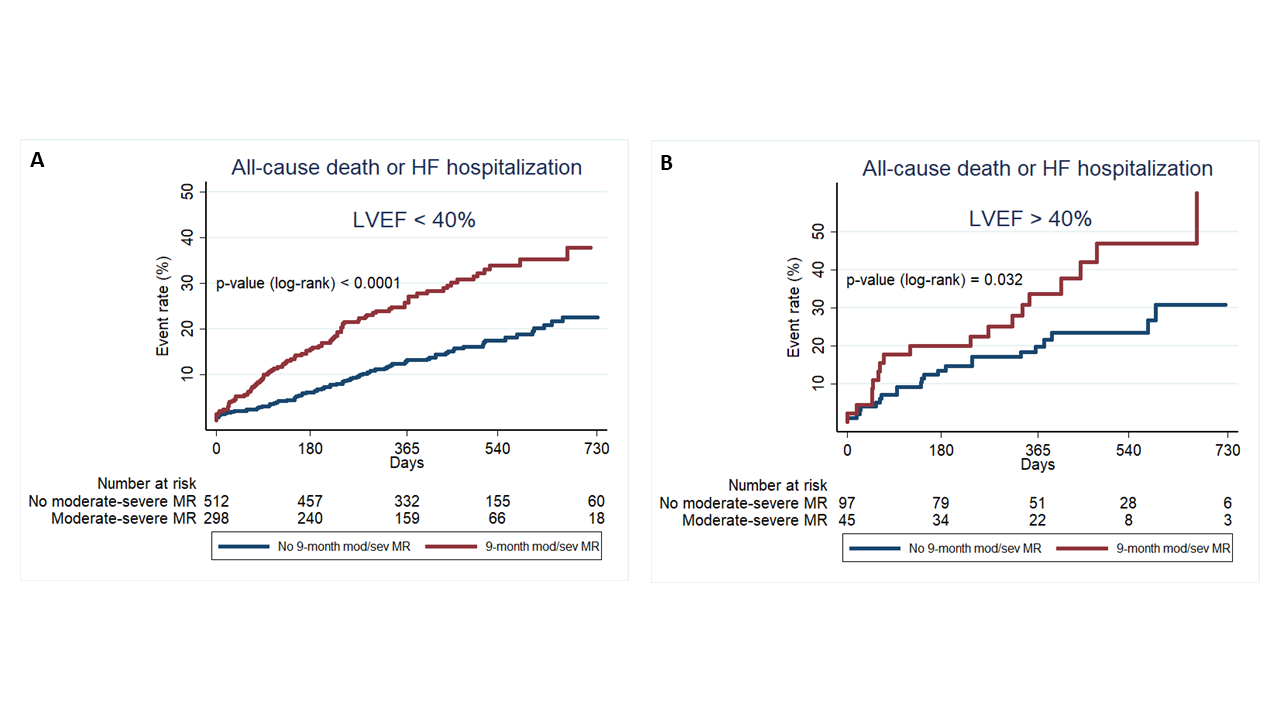


The figure shows Kaplan-Meier curves for 2-year primary endpoint (all-cause mortality or HF hospitalization) in patients with vs. without 9-month moderate-severe MR in the subgroup with LVEF <40% (panel A) and in the subgroup with LVEF ≥40% (panel B).

GRMT = guideline-directed medical therapy; HF = heart failure; LVEF = left ventricular ejection fraction; MR = mitral regurgitation.
